# Serum Trimethylamine N-Oxide Levels Are Associated with Peripheral Artery Disease in Patients with Type 2 Diabetes Mellitus

**DOI:** 10.3390/medicina61122243

**Published:** 2025-12-18

**Authors:** Cing-Yu Liang, Jer-Chuan Li, Chin-Hung Liu, Du-An Wu, Bang-Gee Hsu

**Affiliations:** 1 Department of Internal Medicine, Hualien Tzu Chi Hospital, Buddhist Tzu Chi Medical Foundation, Hualien 97004, Taiwan; 2 Division of Metabolism and Endocrinology, Buddhist Tzu Chi General Hospital, Hualien 97004, Taiwan; 3 Graduate Institute of Clinical Pharmacy, School of Medicine, Tzu Chi University, Hualien 97004, Taiwan; 4 School of Pharmacy, Tzu Chi University, Hualien 97004, Taiwan; 5 School of Medicine, Tzu Chi University, Hualien 97004, Taiwan; 6 Division of Nephrology, Hualien Tzu Chi Hospital, Buddhist Tzu Chi Medical Foundation, Hualien 97004, Taiwan

**Keywords:** trimethylamine N-oxide, peripheral arterial disease, type 2 diabetes mellitus, ankle–brachial index

## Abstract

*Background and Objectives*: Peripheral arterial disease (PAD), frequently observed in individuals with type 2 diabetes mellitus (T2DM), is associated with diminished life quality, increased cardiovascular risk, and higher mortality rates. Similarly, trimethylamine N-oxide (TMAO), a uremic toxin produced by gut microbiota, has been linked to hypertension, cardiovascular disease, and increased overall mortality. In this study, we aimed to investigate whether serum TMAO levels are related to PAD in T2DM cases. *Materials and Methods*: In this cross-sectional investigation performed at one medical center, 120 patients with type 2 diabetes mellitus (T2DM) were included. High-performance liquid chromatography–mass spectrometry and an automated oscillometric device were used to measure serum TMAO levels and ankle–brachial index (ABI) values, respectively. Individuals exhibiting an ABI of less than 0.9 were classified as belonging to the low-ABI group. *Results*: Of the 120 participants, 23 (19.2%) had low ABI. Compared with the normal-ABI group, the low-ABI group was older (*p* = 0.017) and exhibited higher levels of urine albumin-to-creatinine ratio (UACR, *p* < 0.001), C-reactive protein (CRP, *p* < 0.001), and TMAO (*p* < 0.001). After adjusting for age, UACR, and CRP, multivariable logistic regression analysis identified serum TMAO concentration as an independent predictor of PAD in T2DM patients (odds ratio [OR]: 1.051; 95% confidence interval [CI]: 1.017–1.086; *p* = 0.003). In Spearman’s rank correlation analyses, log-transformed left ABI (log-left ABI, *p* = 0.017) and log-right ABI (*p* = 0.001) negatively correlated with log-TMAO. In patients with T2DM, the predictive performance of serum TMAO levels for PAD yielded an area under the receiver operating characteristic (ROC) curve of 0.812 (95% CI: 0.701–0.923; *p* < 0.001). *Conclusions*: Among individuals with T2DM, higher serum TMAO levels were associated with lower left and right ABI values and an increased likelihood of PAD.

## 1. Introduction

As a major global health concern, type 2 diabetes mellitus (T2DM) contributes to disease burden through both its metabolic disturbances and its close association with cardiovascular disease (CVD) [1]. Additionally, chronic hyperglycemia contributes to endothelial dysfunction, oxidative stress, and accelerated atherosclerosis, predisposing patients to macrovascular and microvascular complications [2]. In addition to coronary artery disease and stroke, T2DM is closely associated with peripheral arterial disease (PAD), which reflects underlying systemic atherosclerosis [3]. Hyperglycemia and insulin resistance promote arterial stiffness and impaired collateral circulation, contributing to limb ischemia, delayed wound healing, and increased amputation rates in patients with diabetes mellitus (DM) [4]. The prevalence of PAD is significantly higher among individuals with T2DM than among those without, and its occurrence further heightens the risk of major adverse cardiovascular events and death [5].

Currently, PAD is primarily diagnosed through noninvasive vascular assessments, with the ankle–brachial index (ABI) as its first-line screening tool [6]. For patients whose arteries remain compressible, the ABI continues to serve as a practical and informative tool; however, even in this population, early or subclinical PAD may still be missed because mild or localized atherosclerotic lesions may not produce sufficient hemodynamic changes to lower the ABI, despite the presence of underlying vascular injury [7]. Other diagnostic tools, such as duplex ultrasonography, possess high sensitivity and specificity in providing anatomic and hemodynamic information; however, they are operator-dependent and less effective in assessing diffuse or small-vessel disease [8]. Advanced imaging techniques, such as computed tomographic angiography (CTA) and magnetic resonance angiography (MRA), can provide comprehensive visualization of arterial stenosis and occlusion. However, they are costly, require the use of a contrast agent, and may be contraindicated in patients with renal dysfunction or those with implanted metallic devices [9]. To date, digital subtraction angiography (DSA) is regarded as the definitive diagnostic modality for PAD, offering precise vascular mapping and enabling simultaneous therapeutic intervention [10]. Nevertheless, its invasiveness, risks of developing contrast-induced nephropathy and vascular complications, and unsuitability for routine screening limit its utility [10]. These limitations highlight the need to identify complementary circulating biomarkers that can improve vascular risk stratification in T2DM patients who remain suitable for ABI-based assessment yet may still be misdiagnosed due to early or subclinical PAD, while avoiding the immediate use of more advanced and costly imaging modalities that also carry risks such as contrast-induced nephropathy.

Serum trimethylamine N-oxide (TMAO), a metabolite derived from intestinal microbiota, is formed through the liver oxidation process of trimethylamine (TMA) generated from food-derived precursors such as choline, L-carnitine, and phosphatidylcholine [11]. Elevated circulating TMAO concentrations have been increasingly recognized as a biomarker for adverse cardiovascular outcomes [11]. Mechanistically, TMAO promotes atherosclerosis through multiple pathways, such as enhancing foam cell formation, impairing reverse cholesterol transport, and augmenting platelet hyperreactivity; consequently, the risk of thrombotic events increases [12]. Many studies have shown that elevated TMAO levels are associated with adverse cardiovascular outcomes, and some have reported that higher TMAO concentrations can predict 5-year all-cause mortality in stable patients with PAD [12,13]. However, these studies have not evaluated TMAO as a diagnostic indicator using objective vascular measurements, nor have they examined its relationship with ABI-defined PAD within a well-characterized T2DM population. Compared with ABI, which primarily reflects limb hemodynamics and may remain within the normal range until flow-limiting stenosis is present, TMAO integrates information related to systemic inflammation and metabolic status, potentially capturing earlier or more diffuse vascular injury that is not evident on ABI alone. Hence, this study sought to examine the relationship between serum TMAO concentrations and PAD in individuals with T2DM and to determine whether TMAO could serve as a potential biomarker for vascular risk stratification in this population.

## 2. Materials and Methods

### 2.1. Patients

Between January and June 2020, a cross-sectional study at a tertiary hospital in eastern Taiwan enrolled 120 patients with T2DM. Approval for this research protocol was granted by the Institutional Review Board of Hualien Tzu Chi Hospital, Buddhist Tzu Chi Medical Foundation (IRB No. 110-139-C). Each participant provided written informed consent prior to joining the study. Participants rested in a seated position for at least 10 min before morning blood pressure (BP) assessment, during which systolic blood pressure (SBP) and diastolic blood pressure (DBP) were measured and recorded using a standardized method. Those presenting with ongoing infection, acute coronary syndrome, heart failure, lower extremity amputation, malignant neoplasm, or an ankle–brachial index greater than 1.3, as well as those without informed consent, were not included in the study. Hypertension was defined as SBP ≥ 140 mmHg, DBP ≥ 90 mmHg, or use of antihypertensive medication, whereas DM was defined as fasting blood glucose ≥ 126 mg/dL or treatment with antidiabetic medication.

### 2.2. Anthropometric Analysis and Biochemical Determinations

Anthropometric data, including height and weight, were collected from participants while they were dressed in light clothing and barefoot. The BMI was derived by dividing the weight in kilograms by the square of the height in meters. For sample collection, about 5 mL of fasting venous blood was obtained from each subject and centrifuged at 3000× *g* for 10 min. An automated biochemical analyzer (Advia 1800, Siemens Healthcare GmbH, Erlangen, Germany) was employed to assess serum biomarkers, including fasting blood glucose, glycated hemoglobin (HbA1c), albumin, renal function indicators such as blood urea nitrogen (BUN) and creatinine, lipid profile parameters including total cholesterol, triglycerides, low- and high-density lipoprotein cholesterol (LDL-C and HDL-C), as well as C-reactive protein (CRP). For the determination of the urine albumin-to-creatinine ratio (UACR), on-site spot urine samples were obtained from participants [14]. Finally, participants’ estimated glomerular filtration rate (eGFR) values were calculated using the Chronic Kidney Disease Epidemiology Collaboration (CKD-EPI) formula.

### 2.3. ABI Measurements

Brachial and ankle BP were recorded bilaterally in the supine position using an automated oscillometric device (VaSera VS-1000; Fukuda Denshi, Tokyo, Japan) three times; ABI for each leg was computed as the larger ankle SBP divided by the higher arm SBP, with PAD defined as ABI < 0.9 [15]. Continuous electrocardiographic monitoring was performed for at least 15 min.

### 2.4. Measurement of Serum TMAO Concentration

Quantitative analysis of serum TMAO was performed using a combination of high-performance liquid chromatography and mass spectrometry. The procedure utilized a Waters e2695 Separations Module linked to an ACQUITY QDa quadrupole detector (Waters Corporation, Milford, MA, USA), with trimethylamine-d_9_ N-oxide (d_9_-TMAO) applied as the internal calibration standard. Separation was achieved on a C18 column (5 µm, 250 × 4.6 mm, 100 Å; Phenomenex, Torrance, CA, USA) under an acidified water–methanol gradient at 40 °C, and TMAO/d9-TMAO was monitored in positive-ion mode; chromatographic integration used Empower 3.0 software (Waters Corporation, Milford, MA, USA) as described in detail in a previous report [16].

### 2.5. Statistical Analysis

According to the general principles of observational correlation studies, a sample size of 100–150 participants is recommended to detect moderate effect sizes with adequate statistical power [17]. With an alpha level of 0.05, a sample size of 120 achieved 80% power to detect a correlation coefficient of 0.25 between serum TMAO and PAD. Post hoc power analysis using the observed correlation between log-transformed TMAO (log-TMAO) and log-transformed left ABI values (*r* = 0.218) verified that the sample size afforded approximately 80% power to achieve statistical significance at α = 0.05 [18]. Accordingly, the sample size provided adequate power to detect a moderate relationship between TMAO levels and PAD with reliable precision.

All data processing and statistical analysis were performed using IBM SPSS Statistics version 19.0 (Armonk, NY, USA). Normality of the data distribution was examined using the Kolmogorov–Smirnov test. Data conforming to a normal distribution were shown as mean ± standard deviation, and intergroup differences were tested with the independent-samples *t*-test. For non-normally distributed variables, results were described as medians with interquartile ranges, and intergroup differences were examined using the Mann–Whitney U test. Categorical variables were presented as frequencies (percentages) and compared using the chi-square test. To satisfy the assumptions of parametric analysis, skewed variables were subjected to a logarithmic transformation (base 10). To identify factors independently associated with PAD, a multivariable logistic regression analysis was performed. To identify factors independently associated with PAD, a multivariable logistic regression analysis was performed. Given the limited number of PAD events (*n* = 23), a parsimonious model was selected to prevent overfitting. The model included age, UACR, CRP, and TMAO as covariates, selected based on their physiological relevance and significant associations observed in the univariate analysis. To evaluate the diagnostic accuracy of serum TMAO levels in predicting PAD, receiver operating characteristic (ROC) curve analysis was conducted, and the area under the curve (AUC) was derived using MedCalc version 22.019 (MedCalc Software Ltd., Ostend, Belgium). Meanwhile, associations between log-transformed TMAO, log-transformed left and right ABI values, and physiological parameters were examined using nonparametric Spearman’s rank correlation. Statistical significance was defined as a two-tailed *p*-value below 0.05.

## 3. Results

### 3.1. Participants’ Baseline Characteristics

Table 1 lists the participants’ demographic, clinical, and biochemical information. This study enrolled 120 patients with T2DM, comprising 65 males and 55 females. Of these participants, 70 (58.3%) had hypertension. Common risk factors associated with CVD and atherosclerosis were frequently observed. Furthermore, 23 (19.2%) patients with T2DM had a low ABI. The low-ABI group was older (*p* = 0.017) and had higher CRP (*p* < 0.001), UACR (*p* < 0.001), and TMAO (*p* < 0.001) values than the normal-ABI group. Conversely, BMI, BP, lipid profile, and glycemic control markers showed no significant differences across the two groups. Moreover, the most frequently used medication was dipeptidyl peptidase-4 inhibitor (*n* = 76; 63.3%). Other commonly prescribed medications were sulfonylurea (*n* = 65; 54.2%), metformin (*n* = 64; 53.3%), statins (*n* = 63; 52.5%), insulin (*n* = 29; 24.2%), and fibrates (*n* = 25; 20.8%). Between the low- and normal-ABI groups, no significant differences were noted in sex, associated medical conditions (e.g., hypertension), or the use of lipid-lowering (statins, fibrates) and antidiabetic drugs (metformin, sulfonylureas, DPP-4 inhibitors, and insulin).

### 3.2. Association Between TMAO and PAD

As shown in Table 2, multivariable logistic regression analysis revealed that elevated TMAO levels were independently associated with PAD. In the adjusted model, which controlled for age, UACR, and CRP, serum TMAO remained a significant independent predictor for PAD. For every 1 µg/L increase in TMAO, the risk of PAD increased by 5.1% (OR = 1.051; 95% CI: 1.017–1.086; *p* = 0.003). Additionally, CRP levels were independently associated with PAD (each 0.1 mg/dL increase in CRP, OR = 1.155; 95% CI: 1.012–1.318; *p* = 0.033), whereas age and UACR did not reach statistical significance in the multivariable model. No multicollinearity (variance inflation factors > 10) was noted in this multivariable logistic regression analysis.

### 3.3. Diagnostic Accuracy of TMAO, CRP, UACR, and Age for PAD

**Figure 1:**
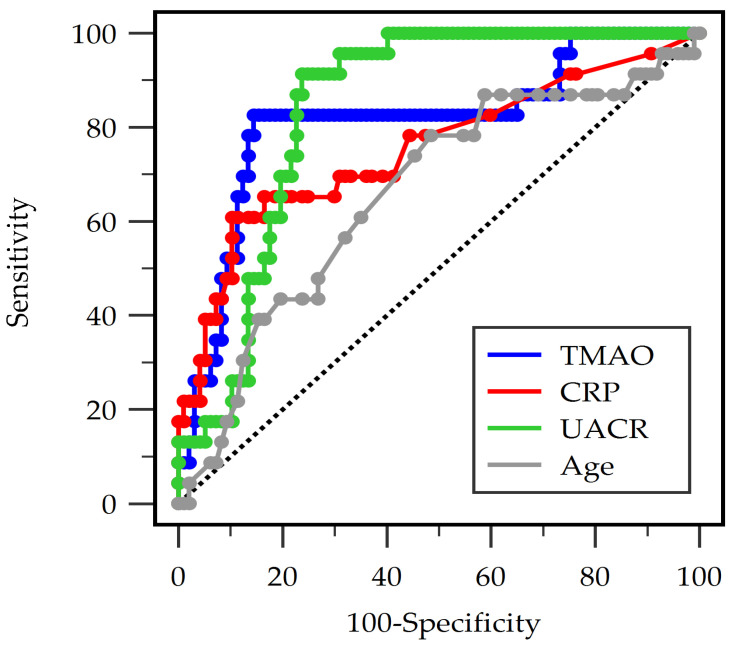
Receiver operating characteristic analysis of trimethylamine N-oxide (TMAO), C-reactive protein (CRP), urine albumin-to-creatinine ratio (UACR), and age to predict peripheral arterial disease in 120 diabetic individuals.

In the ROC analysis, all four variables that were significant in Table 1 of TMAO, CRP, UACR, and age demonstrated significant discriminatory ability for detecting PAD among patients with T2DM (Figure 1). UACR showed the strongest individual performance with an AUC of 0.840 (95% CI: 0.770–0.910; *p* < 0.001), followed by TMAO with an AUC of 0.812 (95% CI: 0.701–0.923; *p* < 0.001). CRP exhibited moderate diagnostic accuracy (AUC 0.754, 95% CI: 0.626–0.881; *p* = 0.0001), whereas age had a lower but still statistically significant AUC of 0.660 (95% CI: 0.532–0.788; *p* = 0.015) (Table 3). The combined model incorporating TMAO, CRP, UACR, and age achieved the highest overall diagnostic performance (AUC 0.864, 95% CI: 0.775–0.953; *p* < 0.001), indicating that integrating multiple biomarkers improves the prediction of PAD beyond the performance of any single variable.

### 3.4. Correlations Between TMAO, ABI, and Clinical Parameters

Spearman correlation analysis (Table 4) showed that log-transformed TMAO (log-TMAO) levels significantly and inversely correlated with both left ABI (*r* = −0.218, *p* = 0.017) and right ABI (*r* = −0.289, *p* = 0.001). TMAO positively correlated with renal dysfunction and inflammation markers such as log-BUN (*r* = 0.340, *p* < 0.001), log-creatinine (*r* = 0.282, *p* = 0.002), log-UACR (*r* = 0.326, *p* < 0.001), and log-CRP (*r* = 0.373, *p* < 0.001). Conversely, TMAO had no significant correlation with BP, glucose, and lipid profile variables. Meanwhile, left and right ABI values negatively correlated with SBP (*r* = −0.257, *p* = 0.005 and *r* = −0.200, *p* = 0.028), log-UACR (*r* = −0.376 and *r* = −0.384, all *p* < 0.001), and log-CRP (*r* = −0.329, *p* < 0.001 and *r* = −0.217, *p* = 0.014). Furthermore, a negative correlation was observed between the right ABI value and total cholesterol (r = −0.233, *p* = 0.010), whereas the left ABI value showed a positive correlation with albumin (r = 0.230, *p* = 0.012).

## 4. Discussion

This study demonstrated that elevated serum TMAO levels were significantly associated with ABI-defined PAD among patients with T2DM whose arteries remained compressible. In addition, patients with T2DM who had lower ABI values exhibited higher concentrations of TMAO, UACR, and CRP, as well as an older age, compared to those with normal ABI values. Therefore, TMAO may be a promising novel biomarker for PAD in T2DM patients, reflecting the mechanistic interplay between metabolites produced by the intestinal microbiota and vascular dysfunction in these patients.

PAD is closely linked to chronic vascular inflammation, which contributes to endothelial dysfunction and the progression of atherosclerotic plaque formation [19]. If arterial inflammation persists, vascular remodeling and luminal narrowing could occur, ultimately impairing blood flow to peripheral tissues [19]. Vascular inflammation in PAD is characterized by immune cell infiltration, activation of inflammatory pathways, and enhanced expression of pro-inflammatory cytokines, such as interleukin-1β and interleukin-6 [20]. Advancing age is also a major risk factor for PAD; the prevalence of this disease markedly increases among older adults [7]. Aging alters the balance of endothelium-derived factors, characterized by the reduced production of vasodilators such as nitric oxide and endothelium-derived hyperpolarizing factors, alongside the increased synthesis of vasoconstrictors, including endothelin-1 and cyclooxygenase-derived prostanoids [21]. These age-related endothelial alterations contribute to vascular dysfunction and play a crucial role in the pathogenesis and progression of PAD [22]. Our findings are consistent with previous reports. For instance, Woleli et al. demonstrated that older individuals with DM have higher PAD incidence [23]. The current study also showed that patients with T2DM exhibiting lower ABI values were significantly older. Furthermore, hypertension facilitates atherosclerosis progression and is linked to an increased incidence and faster progression of PAD [24]. Chronic hypertension promotes vascular remodeling through increased peripheral vascular resistance and arterial stiffness [25]. In individuals with DM, activation of the renin–angiotensin–aldosterone system triggers vasoconstriction, inflammation, and oxidative stress, all leading to the worsening of endothelial dysfunction and the progression of atherosclerosis [26]. According to Lu et al., elevated SBP, as well as DBP ≥90 mmHg, was significantly associated with increased PAD risk [27]. In our study, although the difference in SBP between the normal- and low-ABI groups did not reach statistical significance, the low-ABI group demonstrated a higher SBP value, as determined by Spearman correlation analysis. Additionally, higher CRP and UACR levels were observed in the low-ABI group in the present study. CRP is a well-established marker of systemic inflammation, and elevated levels have been linked to increased incidence, severity, and progression of atherosclerotic disease, including PAD, as well as higher risks of cardiovascular events and mortality [28]. UACR, in turn, reflects renal microvascular damage and generalized endothelial dysfunction, which are particularly relevant in T2DM, where albuminuria has been associated with both macrovascular complications and adverse limb outcomes [29]. Consistent with the conclusions of Kim et al., elevated CRP and UACR levels have been proposed as powerful surrogate markers of more severe PAD and worse prognosis [30]. The concurrent elevation of these biomarkers in the low-ABI group suggests that systemic inflammatory activation and endothelial injury are closely intertwined in the pathophysiology of PAD, supporting the concept that PAD in T2DM is not merely a localized limb disease but a manifestation of widespread vascular dysfunction.

Aside from the traditional risk factors mentioned above, growing evidence indicates that metabolites derived from the intestinal microbiota, particularly TMAO, are key contributors to endothelial dysfunction, cardiovascular events, and impaired limb perfusion in patients with DM [31,32,33]. TMAO is a small organic compound generated in the human body through a two-step metabolic pathway. In the first step of TMAO biosynthesis, the intestinal microbiota metabolize dietary precursors—chiefly choline and L-carnitine—into trimethylamine (TMA) via microbial enzymes including choline TMA-lyase and carnitine monooxygenase. Subsequently, TMA enters the portal circulation and is delivered to the liver, where it undergoes oxidation to TMAO catalyzed by the hepatic enzyme flavin-containing monooxygenase 3 (FMO3) [11,12]. Gut microbiota dysbiosis is more pronounced in patients with DM, characterized by reduced microbial diversity and altered bacterial composition, than in healthy individuals [34]. Furthermore, dysbiosis in such patients exhibits reduced microbial diversity, depletion of bacteria that produce short-chain fatty acids, and an enrichment of TMA-producing genera, including Prevotella and certain members of the Ruminococcaceae and Lachnospiraceae families [35,36]. This shift favors enhanced microbial metabolism of choline and carnitine to TMA, thereby increasing substrate availability for hepatic FMO3 to generate TMAO [37]. According to the previous meta-analysis and systematic review, Farhangi et al. reported a dose-dependent relationship between TMAO concentration and CRP level, with patients with DM in the highest TMAO category exhibiting significantly higher CRP levels [38]. This finding is similar to that of the present study, in which serum TMAO levels were positively correlated with CRP values. Additionally, consistent with our current results, Kalagi et al. revealed that plasma TMAO levels are significantly associated with chronic kidney disease, and this association was further aggravated in individuals with DM; such TMAO levels also correlated with renal function biomarkers, including serum creatinine, eGFR, BUN, and UACR [39]. TMAO is metabolized and excreted through the kidneys; therefore, impaired renal function can lead to elevated circulating TMAO levels [40]. In accordance with mechanistic insights, the presented study demonstrated that elevation of serum TMAO concentrations was significantly associated with systemic inflammation, reflected by higher CRP levels, and with renal dysfunction, evidenced by increased BUN, creatinine, and UACR levels, together with a decline in eGFR. These findings support the notion that in DM cases, renal impairment may act synergistically to elevate TMAO levels, thereby enhancing systemic inflammation through nontraditional pathways.

TMAO is reportedly a potential biomarker of inflammation and endothelial dysfunction, with substantial evidence supporting its association with vascular pathologies, including PAD [41,42]. Roncal et al. conducted a multivariate analysis and found that plasma TMAO levels increased in proportion to PAD severity and were significantly associated with an elevated risk of cardiovascular mortality [41]. Several studies have reported that elevated TMAO levels are associated with accelerated atherosclerosis, endothelial dysfunction, and increased platelet reactivity, and that higher TMAO concentrations can predict 5-year all-cause mortality among patients with PAD; however, these findings relate primarily to prognosis and have not established TMAO as a diagnostic indicator or evaluated its association with ABI-defined PAD in a clearly characterized T2DM population [12,13,32,33]. Our study extends the association beyond traditional risk factors; patients with T2DM exhibiting lower ABI values had higher circulating TMAO concentrations than those with normal ABI. Despite adjustment for key physiological confounding factors and statistical significance in Table 1 (age, UACR, CRP, and TMAO), our multivariate logistic regression analysis confirmed that elevated serum TMAO levels were an independent and significant positive predictor of PAD. These findings are in agreement with an expanding body of literature suggesting that TMAO is more than a passive biomarker of vascular injury; it actively participates in pathogenic processes leading to vascular dysfunction and damage. As reported by Sun et al. [43], TMAO elevates reactive oxygen species (ROS) levels, leading to the activation of thioredoxin-interacting protein (TXNIP) and the subsequent initiation of the NLR family pyrin domain-containing 3 (NLRP3) inflammasome, thereby promoting vascular inflammation. Consequently, this inflammatory activation promotes the release of pro-inflammatory cytokines, including interleukin-1β and interleukin-18, while simultaneously impairing endothelial function through decreased nitric oxide synthase activity and reduced nitric oxide bioavailability. In addition, Chen, L. et al. reported that increased TMAO levels impair perfusion recovery and angiogenesis in experimental PAD [32]. These alterations lead to impaired vasorelaxation, weakened perfusion recovery, and diminished angiogenesis, all of which play pivotal roles in PAD pathophysiology [32,43]. Based on prior research and our findings, the observation that TMAO independently correlates with lower ABI is consistent with growing evidence showing that TMAO not only predicts poorer prognosis in patients with PAD but also contributes to vascular dysfunction in the T2DM population [12,13,32,33]. While ABI primarily reflects limb hemodynamics and may remain within the normal range until flow-limiting stenosis develops, TMAO reflects systemic inflammatory activation and endothelial injury, both of which are central to the early pathogenesis of PAD. These biological characteristics suggest that TMAO may serve as a complementary diagnostic indicator, helping to identify early or diffuse vascular impairment that may not be readily detected by ABI alone.

Although this study demonstrated that higher TMAO levels were associated with PAD in patients with T2DM, some limitations still exist. First, the cross-sectional design limits causal inference, and the insight into whether the increased production of TMAO facilitates PAD development or is simply a manifestation of PAD severity remains unclear. Second, the diet pattern, gut microbiome composition, use of other medications, and lifestyle factors of the participants were not assessed. Thus, the results may be affected by potential confounding factors for the observed relations. Third, our study relied solely on resting ABI without adjunctive duplex ultrasonography, CTA/MRA, or DSA, which may lead to disease misclassification, particularly in individuals with medial arterial calcification or small-vessel involvement. Although patients with an ABI > 1.30 were excluded to minimize non-compressibility-related misclassification, this approach further narrows the sample to T2DM patients with compressible arteries, potentially underestimating the true prevalence of PAD. Consequently, our findings cannot be generalized to individuals with non-compressible arteries, in whom ABI is least reliable. Finally, the study has a relatively small sample size and was conducted in only one institution, thereby potentially limiting the generalizability of the results. Specifically, the absolute number of PAD events was low (n = 23). Although we employed a multivariable model to adhere to statistical rules of thumb, the small number of events relative to potential predictors limits the ability to extensively adjust for all metabolic covariates without risking model overfitting. Thus, multiple prospective, multicenter studies with larger sample sizes are warranted to confirm the relationship between serum TMAO levels and PAD in patients with T2DM.

## 5. Conclusions

Serum TMAO concentrations demonstrated significant negative correlations with both left and right ABI measurements and were independently associated with the occurrence of PAD among individuals with T2DM. Importantly, these findings apply specifically to individuals with T2DM and compressible arteries. TMAO levels were also correlated with markers of systemic inflammation and endothelial dysfunction, suggesting that higher concentrations may reflect broader vascular injury processes. In contrast to ABI, which primarily captures limb hemodynamics, TMAO reflects systemic inflammatory and endothelial injury pathways that may precede overt hemodynamic compromise. Together, these properties suggest that TMAO may serve as a complementary marker alongside ABI, providing additional biological information that could enhance vascular risk stratification and support the earlier identification of PAD in individuals with T2DM. Further research with larger cohorts and comprehensive statistical analyses is needed to validate these results and determine the clinical applicability of TMAO in routine practice.

## Figures and Tables

**Table 1:** Clinical variables of 120 diabetic patients were compared with those of patients with a normal or low ankle–brachial index (<0.9).

Characteristic	All Patients (*n* = 120)	Control Group (*n* = 97)	Low-ABI Group (*n* = 23)	*p* Value
Age (years)	66.00 (57.25–71.00)	64.00 (56.50–70.00)	68.00 (65.00–75.00)	0.017 *
Height (cm)	161.10 ± 8.61	161.84 ± 8.73	157.98 ± 7.46	0.053
Body weight (kg)	69.98 ± 13.86	70.71 ± 13.94	66.93 ± 13.39	0.242
Body mass index (kg/m^2^)	26.84 ± 4.09	26.89 ± 4.11	26.66 ± 4.10	0.816
Left-ankle–brachial index	1.06 (1.00–1.12)	1.07 (1.03–1.13)	0.89 (0.84–0.94)	<0.001 *
Right-ankle–brachial index	1.07 (1.02–1.13)	1.10 (1.05–1.15)	0.91 (0.88–0.96)	<0.001 *
SBP (mmHg)	143.95 ± 20.66	142.64 ± 20.32	149.48 ± 21.63	0.154
DBP (mmHg)	82.91 ± 11.05	82.94 ± 10.52	82.78 ± 13.32	0.952
Albumin (mg/dL)	4.24 ± 0.26	4.25 ± 0.26	4.22 ± 0.27	0.591
Total cholesterol (mg/dL)	162.13 ± 33.18	160.27 ± 30.88	170.00 ± 41.40	0.207
Triglyceride (mg/dL)	122.00 (84.25–170.50)	122.00 (84.00–171.00)	101.00 (84.00–171.00)	0.957
HDL-C (mg/dL)	46.97 ± 15.58	46.63 ± 16.37	48.39 ± 11.88	0.628
LDL-C (mg/dL)	98.61 ± 28.93	98.39 ± 28.23	99.52 ± 32.38	0.867
Fasting glucose (mg/dL)	137.50 (121.00–168.75)	136.00 (121.00–165.00)	138.00 (121.00–196.00)	0.610
HbA1c (%)	7.40 (6.60–8.65)	7.30 (6.60–8.40)	8.10 (6.70–9.40)	0.553
Blood urea nitrogen (mg/dL)	16.00 (12.00–19.00)	15.00 (12.00–18.00)	18.00 (12.00–22.00)	0.265
Creatinine (mg/dL)	0.90 (0.70–1.10)	0.90 (0.70–1.10)	0.80 (0.60–0.90)	0.206
eGFR (mL/min)	85.11 ± 30.03	84.68 ± 30.25	86.90 ± 29.66	0.751
UACR (mg/g)	27.39 (9.96–160.62)	19.94 (8.73–66.58)	179.93 (109.73–459.32)	<0.001 *
C-reactive protein (mg/dL)	0.11 (0.08–0.31)	0.09 (0.08–0.20)	0.44 (0.11–1.26)	<0.001 *
TMAO (μg/L)	20.82 (14.19–31.31)	19.72 (13.44–25.41)	37.92 (30.64–55.62)	<0.001 *
Male, *n* (%)	65 (54.2)	56 (57.7)	9 (39.1)	0.107
Current smoking, *n* (%)	11 (9.2)	8 (8.2)	3 (13.0)	0.474
Hypertension, *n* (%)	70 (58.3)	53 (54.6)	17 (73.9)	0.092
ACE inhibitor use, *n* (%)	11 (9.2)	9 (9.2)	2 (8.7)	0.931
ARB use, *n* (%)	59 (49.2)	45 (46.4)	14 (60.9)	0.212
β-blocker use, *n* (%)	13 (10.8)	10 (10.3)	3 (13.0)	0.704
CCB use, *n* (%)	30 (25.0)	24 (24.7)	6 (26.1)	0.893
Statin use, *n* (%)	63 (52.5)	51 (52.6)	12 (52.2)	0.972
Fibrate use, *n* (%)	25 (20.8)	20 (20.6)	5 (21.7)	0.905
Metformin use, *n* (%)	64 (53.3)	52 (53.6)	12 (52.2)	0.901
Sulfonylurea use, *n* (%)	65 (54.2)	51 (52.6)	14 (60.9)	0.473
DDP-4 inhibitor use, *n* (%)	76 (63.3)	60 (61.9)	16 (69.6)	0.490
Insulin use, *n* (%)	29 (24.2)	24 (24.7)	5 (21.7)	0.762
SGLT2i use, *n* (%)	24 (20.0)	19 (19.6)	5 (21.7)	0.817

Continuous variables are expressed as mean ± standard deviation and compared using Student’s *t*-test; non-normally distributed variables are presented as median and interquartile range and compared using the Mann–Whitney *U* test; categorical variables are expressed as number (%) and were compared using the chi-square test. ABI, ankle–brachial index; SBP, systolic blood pressure; DBP, diastolic blood pressure; HDL-C, high-density lipoprotein cholesterol; LDL-C, low-density lipoprotein cholesterol; HbA1c, glycated hemoglobin; eGFR, estimated glomerular filtration rate; UACR, urine albumin-to-creatinine ratio; TMAO, Trimethylamine N-oxide; ACE, angiotensin-converting enzyme; ARB, angiotensin receptor blocker; CCB, calcium channel blocker; DDP-4, dipeptidyl peptidase 4; SGLT2i, sodium–glucose cotransporter 2 inhibitors. * *p* < 0.05 was considered statistically significant.

**Table 2:** Multivariable logistic regression analysis of the factors correlated to peripheral arterial disease among 120 patients with type 2 diabetes mellitus.

Variables	Odds Ratio	95% Confidence Interval	*p* Value
TMAO, 1 μg/L	1.051	1.017–1.086	0.003 *
C-reactive protein, 0.1 mg/dL	1.155	1.012–1.318	0.033 *
Age, 1 year	1.031	0.982–1.082	0.225
UACR, 1 mg/g	1.001	0.999–1.002	0.263

The analysis data was performed using the multivariable logistic regression analysis (adopted factors: age, UACR, C-reactive protein, and TMAO). UACR, urine albumin-to-creatinine ratio; TMAO, Trimethylamine N-oxide. * *p* < 0.05 by multivariable logistic regression analysis.

**Table 3:** The area under the receiver operating characteristic curve analysis for trimethylamine N-oxide, C-reactive protein, urine albumin-to-creatinine ratio, age, and combined variables in predicting peripheral arterial disease among 120 diabetic patients.

Variables	AUC	95% Confidence Interval	*p* Value
TMAO	0.812	0.701—0.923	<0.001 *
CRP	0.754	0.626—0.881	0.0001 *
UACR	0.840	0.770—0.910	<0.001 *
Age	0.660	0.532—0.788	0.015 *
TMAO + CRP + UACR + age	0.864	0.775—0.953	<0.001 *

AUC, area under the receiver operating characteristic curve; CRP, C-reactive protein; UACR, urine albumin-to-creatinine ratio. * *p* < 0.05 was considered statistically significant.

**Table 4:** Spearman correlation coefficients showing the relationships between left ABI, right ABI, log-TMAO, and clinical variables among 120 diabetic patients.

Variables	Log-Left ABI		Log-Right ABI		Log-TMAO (μg/L)	
	Spearman Coefficient of Correlation	*p* Value	Spearman Coefficient of Correlation	*p* Value	Spearman Coefficient of Correlation	*p* Value
Log-Age (years)	–0.135	0.141	–0.051	0.582	0.134	0.144
Body mass index (kg/m^2^)	–0.039	0.678	–0.050	0.586	–0.130	0.156
Log-left ABI	—	—	0.628	<0.001 *	–0.218	0.017 *
Log-right ABI	0.628	<0.001 *	—	—	–0.289	0.001 *
Log-TMAO (μg/L)	–0.218	0.017 *	–0.289	0.001 *	—	—
SBP (mmHg)	–0.257	0.005 *	–0.200	0.028 *	0.015	0.873
DBP (mmHg)	–0.002	0.987	–0.007	0.943	0.009	0.924
Albumin (mg/dL)	0.230	0.012 *	0.168	0.067	–0.160	0.081
Total cholesterol (mg/dL)	–0.165	0.072	–0.233	0.010 *	0.068	0.462
Log-Triglyceride (mg/dL)	–0.035	0.704	–0.009	0.924	0.091	0.324
HDL-C (mg/dL)	–0.089	0.336	–0.165	0.071	0.004	0.969
LDL-C (mg/dL)	–0.072	0.433	–0.156	0.088	–0.094	0.309
Log-Glucose (mg/dL)	0.119	0.194	–0.007	0.936	0.102	0.268
Log-HbA1c (%)	–0.012	0.893	–0.113	0.221	0.104	0.260
Log-BUN (mg/dL)	–0.094	0.305	–0.130	0.158	0.340	<0.001 *
Log-Creatinine (mg/dL)	0.031	0.740	0.066	0.474	0.282	0.002 *
eGFR (mL/min)	0.068	0.460	0.002	0.981	–0.236	0.009 *
Log-UACR (mg/g)	–0.376	<0.001 *	–0.384	<0.001 *	0.326	<0.001 *
Log-CRP (mg/L)	–0.329	<0.001 *	–0.217	0.014 *	0.373	<0.001 *

Variables, including age, TMAO, ABI, triglycerides, glucose, HbA1c, BUN, creatinine, UACR, and CRP, were log-transformed due to their skewed distributions. Data analysis was conducted using the nonparametric Spearman’s rank correlation method. TMAO, trimethylamine *N*-oxide; ABI, ankle–brachial index; SBP, systolic blood pressure; DBP, diastolic blood pressure; HDL-C, high-density lipoprotein cholesterol; LDL-C, low-density lipoprotein cholesterol; HbA1c, glycated hemoglobin; BUN, blood urea nitrogen; eGFR, estimated glomerular filtration rate; UACR, urine albumin-to-creatinine ratio; CRP, C-reactive protein. * *p* < 0.05 was considered statistically significant (two-tailed).

## Data Availability

Upon request, the corresponding author can provide the data utilized in this study.

## Abbreviations

The following abbreviations are used in this manuscript:

ABI	ankle–brachial index
aOR	adjusted odds ratio
AUC	area under the curve
BP	blood pressure
BUN	blood urea nitrogen
CI	confidence interval
CTA	computed tomographic angiography
CVD	cardiovascular disease
CRP	C-reactive protein
DBP	diastolic blood pressure
DM	diabetes mellitus
DSA	digital subtraction angiography
eGFR	estimated glomerular filtration rate
HbA1c	glycated hemoglobin
MRA	magnetic resonance angiography
OR	odds ratio
ROC	receiver operating characteristic
SBP	systolic blood pressure
T2DM	type 2 diabetes mellitus
TMAO	trimethylamine N-oxide
UACR	urine albumin-to-creatinine ratio
